# Some Plans, Practical and Otherwise

**Published:** 1915-10-23

**Authors:** 


### TEMPORARY HOSPITALS' CONSTRUCTION.

### SOME PLANS, PRACTICAL AND OTHERWISE.

We have thought it of historic interest, as well as of first importance as a guide to those who may be concerned in the planning of temporary buildings for the provision of hospital beds for the wounded, to give types of the methods and plans of schemes of this description. They include the addition of temporary buildings in connection with the General Hospital at Nottingham, a block plan of the 3rd London General Hospital, Wandsworth, S.W., containing 500 beds, and a block plan with details of the open-air pavilions, being the latest extension of the 5th Northern General Hospital, Leicester. A study of the plans will enable the technical reader to grasp at once the relative efficiency or otherwise of these various plans, of which we publish a brief detailed description in each case. We have only space here to point out that the plan pursued in connection with the General Hospital, Nottingham, like that of the recent addition at great cost of a new operation theatre unit to the Taunton and Somerset Hospital, Taunton, is to be avoided. We hope nothing of the kind will ever be repeated in connection with the planning of hospital extensions or new construction in this country. (See plans, pp. 72 and 78.)

### A Plan Full of Instruction.

In the case of the 3rd London General Hospital the block plan will show many things which it is essential to avoid. This hospital, from the nature of the site, cannot fulfil the initial conditions which are required to make a temporary hospital for the treatment and care of wounded sailors and soldiers as efficient and complete as it ought to be. One cannot get more than a pint into a pint pot. To crowd pavilions together in order to provide a great number of beds under conditions lower than the best standard is to pursue a policy ill-calculated to promote the complete recovery of, and the best results to the patients whose speedy return to the Colours in full health and in the greatest numbers is surely the first consideration to bear in mind when planning and sanctioning the erection of a hospital of this type. Of course, a large hospital like the 3rd London General Hospital, situated in the centre of a great city, can only be used as a hospital pure and simple, and must, to prove effective, be connected with ample convalescent accommodation of the best type, to which patients can be transferred the moment they are in a position to leave the hospital.

### Some Practical Suggestions.

The 5th Northern General Hospital, Leicester, on the whole presents some of the best features of a military hospital in war-time, for it is able to fulfil fairly completely the requirements on which we have insisted. It is desirable that the worst wards in the converted buildings on the ground floor shall have the old windows removed and louvre windows substituted without delay. The cost of this will be trifling, but the results to its hygienic condition cannot fail to prove of importance. The open-air type of wards which constitute the latest extension to this hospital have proved excellent in summer time. Providing ample bedding is forthcoming, and the blind and shutter shelters are kept in order and utilised to the full, they should prove satisfactory and advantageous in winter time too. Their success or failure in the latter season will depend upon the administration of the wards being of the best. These criticisms apply especially to the most exposed portions of these wards. They hold good to a less extent when applied to the whole five blocks containing 104 beds each, though only a relatively small number of the beds are open to full exposure from wind and weather. Upon the commandant and the matron rests the great responsibility of impressing upon the staff, and seeing that their instructions are carried out, as to how and when the blinds and shutters are to be used in varying circumstances. Care must be taken to see that the night sisters or superintendents do not interfere with the closing of the shutters or the drawing down of the blinds after sundown when they comply with the regulations of the commandant or matron; otherwise in certain circumstances serious trouble may result to some of the patients. These wards are essentially a form of construction which demands constant supervision on the part of the higher officials. They do away with the provision of verandahs, and are an alternative to the taking up of greater space by ward blocks which would then be provided with verandahs. Where the verandahs have been large enough to accommodate all the beds in a ward it has happened that the wards have again been re-bedded and filled with patients, a plan which is wholly indefensible in the hygienic sense. The Leicester experiment is therefore one to be studied.

### The Open-Air Type of Wards.

After the necessary experience it will be possible to give a reliable opinion as to the relative merits and advantages of the open-air type of wards compared with ward blocks with ample verandahs of the type referred to.

We could have wished that the nurses' cloakroom and lavatory had been placed on a less conspicuous position on the site. The blocks of open-air wards are situated quite away and distinct from the old hospital buildings; each has an abundance of sunlight, and the appearance they present in whole and in part is attractive and satisfactory. The nearly flat roofs are a feature, as will be seen from the illustration of them which we give (page 72). The patients, we gathered from our inquiries, have not unnaturally preferred so far the open-air wards, and this preference is likely to continue if the steps we have advised are consistently and invariably followed in wet and unfavourable weather.

